# Texture analysis can predict response to etoposide-doxorubicin-cisplatin in patients with adrenocortical carcinoma

**DOI:** 10.1007/s40618-024-02476-2

**Published:** 2024-10-09

**Authors:** Filippo Crimì, Francesca Turatto, Carlo D’Alessandro, Giovanni Sussan, Maurizio Iacobone, Francesca Torresan, Irene Tizianel, Cristina Campi, Emilio Quaia, Mario Caccese, Filippo Ceccato

**Affiliations:** 1 Department of Medicine-DIMED, University of Padova, Padova, Italy; 2 Institute of Radiology, University-Hospital of Padova, Padova, Italy; 3 Endocrine Surgery Unit, Department of Surgery, Oncology, and Gastroenterology, University of Padova, Padova, 35128 Italy; 4 Endocrinology Unit, Department of Medicine-DIMED, University of Padova, Via Ospedale Civile, Padova, 105 - 35128 Italy; 5 Department of Mathematics, University of Genoa, Genoa, Italy; 6 Life Science Computational Laboratory, Istituto di Ricovero e Cura a Carattere Scientifico Ospedale Policlinico San Martino, Genoa, Italy; 7 Department of Oncology, Oncology 1, Veneto Institute of Oncology IOV-IRCCS, Padua, Italy

**Keywords:** Radiological imaging, Adrenocortical carcinoma (ACC), Adrenal pathology, Radiomics, Image-based texture

## Abstract

**Background:**

The adrenocortical carcinoma (ACC) is a rare and highly aggressive malignancy originating from the adrenal cortex. These patients usually undergo chemotherapy with etoposide, doxorubicin, cisplatin and mitotane (EDP-M) in case of locally advanced or metastatic ACC. Computed tomography (CT) radiomics showed to be useful in adrenal pathologies. The study aimed to analyze the association between response to EDP-M treatment and CT textural features at diagnosis in patients with locally advanced or metastatic ACCs.

**Methods:**

We enrolled 17 patients with advanced or metastatic ACC who underwent CT before and after EDP-M therapy. The response to treatment was evaluated according to RECIST 1.1, Choi, and volumetric criteria. Based on the aforementioned criteria, the patients were classified as responders and not responders. Textural features were extracted from the biggest lesion in contrast-enhanced CT images with LifeX software. ROC curves were drawn for the variables that were significantly different (*p* < 0.05) between the two groups.

**Results:**

Long-run high grey level emphasis (LRHGLE_GLRLM) and histogram kurtosis were significantly different between responder and not responder groups (*p* = 0.04) and the multivariate ROC curve combining the two features showed a very good AUC (0.900; 95%IC: 0.724-1.000) in discriminating responders from not responders. More heterogeneous tissue texture of initial staging CT in locally advanced or metastatic ACC could predict the positive response to EDP-M treatment.

**Conclusions:**

Adrenal texture is able to predict the response to EDP-M therapy in patients with advanced ACC.

**Supplementary Information:**

The online version contains supplementary material available at 10.1007/s40618-024-02476-2.

## Introduction

Adrenocortical carcinoma (ACC) is a rare malignant neoplasm originating from the adrenal cortex, with an estimated incidence of 0.5 to 2 new cases per million annually [1–3]. Diagnosis relies on the contemporary and interdisciplinary evaluation of clinical, endocrine, and imaging assessments before treatment (either surgery or chemotherapy). Prognostic evaluation is challenging, because overall and progression-free survival is often poor, especially in advanced disease [4], and its complex management requires a multidisciplinary evaluation [5].

The only considered curative strategy for ACC is the total resection of the tumor mass [3], combined with adjuvant mitotane in selected patients with a high risk of recurrence [6]. In case of recurrent ACC in patients treated with mitotane, or in those with advanced or metastatic disease at presentation, etoposide-doxorubicin-cisplatin (EDP) therapy combined with mitotane (EDP-M) is the recommended treatment [7]. Actually, there are no radiological factors that can predict the response to EDP-M.

Radiomics, an emerging field of radiology in recent years, uses the analysis of texture in biomedical images to provide quantitative parameters that may be useful as diagnostic support in oncology. Radiomics utilizes the vast amount of data obtained from the latest diagnostic techniques, processes it, and derives multiple quantitative parameters, enabling automated analyses even for highly heterogeneous lesions [8, 9]. Primary masses, as well as large liver or lung metastasis, are often heterogeneous, with necrosis, increased vascularization, colliquation, and locally invasive behavior. In oncology, tissue heterogeneity is associated with variations in genomic subtypes, expression of growth and angiogenic factors, and alterations in the tumor microenvironment, translating into regional variations within individual tumors in terms of proliferation, cell death, metabolic activity, and vascularization [10].

In clinical practice, tumor heterogeneity can be evaluated using histological or imaging data. In the last years, the assessment of heterogeneity through imaging has been limited to qualitative and subjective evaluations by the reporting physician [11]. In this context, radiomic analysis of tumor texture has recently emerged, being able to conduct a non-invasively quantification of heterogeneity, through CT texture analysis. Radiomic parameters can predict cancer behavior, its response to treatment, guiding prognosis in different settings, including head and neck, esophagus, lung, breast, and colorectal tumors [12–18].

In adrenal pathology, several studies have recently demonstrated how radiomics can be crucial in discriminating between benign and malignant adrenal masses, especially in those contexts where the examined mass does not fit into the conventional dimensional and attenuation criteria that could reasonably exclude carcinomatous characteristics [19–22].

At the best of our knowledge, there are no studies in patients with advanced ACC that correlate pre-treatment texture information with anti-tumor therapy outcome. The present study aims to determine whether texture parameters derived from enhanced CT scans of the target lesion with larger dimensions in patients with metastatic/advanced ACC pre-treatment with EDP-M regimen are associated with the response.

## Materials and methods

### Patients

This retrospective observational study included patients with advanced or metastatic adrenocortical carcinoma who underwent EDP-M therapy between 2007 and 2022. The inclusion criteria were: histological diagnosis of ACC or, if not available, endocrine secretion and radiological features indicative of ACC; locally advanced or metastatic disease stage; patients who meet the criteria for EDP-M chemo-therapy defined by ENS@T; availability in of both contrast-enhanced chest and abdominal CT before and after EDP-M (from 4 to 6 cycles); measurable disease tissue load in CT.

A patient was candidate to EDP therapy according to the Guidelines: adults with advanced ACC not amenable to radical resection, irrespective of debulking surgery, or those with progressive or recurrent disease after mitotane monotherapy, or with early (less than six months) recurrence after first-line treatment [3]. The response was evaluated in the first imaging after the EDP-M cycle. EDP-M was administered according to FIRM-ACT study: etoposide 100 mg/m^2^ (day 2–3–4), doxorubicin 40 mg/m^2^ (day 1), cisplatin 40 mg/m^2^ (day 3–4) [7]. Mitotane levels were assessed two-four weeks after its initiation, then every 8 to 12 weeks (or in case of suspected adverse events) with the Lysosafe service database (HRA Pharma, France) [23]. The exclusion criteria were: coarse moving artifacts; measurable disease tissue load; CT scans without thorax and abdomen venous phase; CT scans performed over an acceptable time frame of three months from the start of therapy and the end of therapy; incomplete EDP-M therapy (less than four cycles).

The Ethic Committee of the Istituto Oncologico Veneto approved the study (CET ANV 2023 − 120), and all patients gave their written informed consent to their data being used for research. The database is available in the repository of the University of Padova, depicted in references [24].

### CT imaging procedure

A 64-slice CT scanner (Somatom Sensation, Siemens Healthineers, Erlangen, Germany) was used for imaging. The protocol included unenhanced, arterial (15 s after the achievement of 100 HU within the abdominal aorta lumen), and venous-phase (80 s after intravenous contrast injection) acquisitions after intravenous injection of 2 mL/kg of iohexol 350 mg I/mL (Omnipaque, GE Healthcare, Milwaukee, WI, USA) followed by a 50 mL saline flush. In some cases, a late image acquisition 3 min after contrast injection was performed. The slice thickness reconstruction was 5 mm for unenhanced scans and 3 mm for arterial, venous, and late phases scans.

### Image analysis and processing

CT images were analyzed independently by two radiologists with 5 and 15 years of experience in abdominal oncological imaging.

In each patient, one to three target tumoral lesions were identified, whether they were advanced primary adrenal lesions or metastatic abdominal or thoracic lesions. Each lesion was measured according to the two purely dimensional criteria RECIST 1.1 and 3D-EPSSG, as well as by hybrid Choi, dimensional, and attenuative criteria:

- According to the RECIST 1.1 guidelines [25], the largest diameter has been identified on any available plane (axial, coronal or sagittal). In the post-treatment evaluation, the maximum diameter was measured on the same plane considered at baseline, but not necessarily on the same level of the slice or in the same direction.
- When considering the volumetric evaluation with the 3D-EpSSG criteria, the three maximum diameters of a tumor were evaluated according to the guidelines of the 3D-EpSSG RMS 2005 protocol [26]. The two maximum perpendicular diameters were evaluated in the axial plane on the section with the largest tumor surface while the skull-caudal dimension was measured on sagittal or coronal images. The tumor volume was then obtained by the following formula: a x b x c x π/6, which approximates the geometry of the tumor to an ellipsoid.
- The evaluation according to the Choi criteria assumes that the attenuation values of tumor lesions can give additional information. Hence, we considered both the largest diameter of the lesion on any plane and the mean attenuation value in the venous scan [27].

To facilitate comparison between methods, 3D-EPSSG’s exclusive response sub-categories such as MPR (minor partial response) and very good partial response (VGPR) have been considered as partial response (PR) (Table 1). A comparison of the agreement between the measurements of the two radiologists was performed. After that, the evaluations of the most expert reader were used for the following parts of the study.

**Table 1:** Decision criteria for assignment to response or non-response categories based on RECIST 1.1, 3D-EPSSG and Choi criteria

Response category	1D RECIST 1.1	3D-EPSSG	Choi
Complete response (CR)	Disappearance of all target lesions or lymph node short axis < 10 mm	Disappearance of all target lesions	Disappearance of all target lesions
Partial response (PR)	Reduction > 30% in the sum of the largest diameters of the target lesions	Mass reduction in a range between ≥ 33% and < 100%	Reduction ≥ 10% of tumor dimensions or attenuation (HU) reduction ≥ 15%; no new lesions
Stable disease (SD)	Does not fit into any other category	Does not fit in PR or PD	Does not fit into any other category
Progressive disease (PD)	At least a 20% increase in the sum of target lesion diameters, taking the smallest sum in the study as the reference. In addition to the 20% relative increase, the sum must also demonstrate an absolute increase of at least 5 mm. (Note: the appearance of one or more new lesions is also considered progression)	increase ≥ 40%	increase ≥ 10% in the sum of the largest diameters of the target lesions; does not fit in PR for attenuation (HU); intratumoral nodules occurrence or increase of the pre-existing ones

For the evaluation of the patients’ response to chemotherapy, the best overall response among the lesions was considered. In the light of the study by Ambrosini et al. [28], each patient was classified as a responder to chemotherapy if at least a partial response in all the three criteria mentioned above was achieved, otherwise the patient was classified as a non-responder.

Finally, the largest lesion identified at staging was chosen for each patient and was analyzed through the LIFEx program, a freeware developed for texture analysis [29]. The two radiologists above mentioned in consensus manually delineated a Region of Interest (ROI) around the boundaries of the lesion in each axial slice of the venous phase. Thus, a volume of interest (VOI) of the largest lesion was obtained. For the texture analysis, a resampling of images TC images to a voxel of 3 × 1 × 1 mm was performed. Texture analysis was conducted separately for each lesion using LifeX’s analysis algorithms that generated 104 first and second order features.

### Statistical analysis

The degree of agreement between the quantitative (measured size of the lesions) and qualitative assessments of the two radiologists (namely the assignment to the response categories with the three criteria) was evaluated by the calculation of the weighted Kappa of Cohen. The features obtained from the LIFEx software processing were compared between the two groups of responder and non-responder to chemotherapy. The comparison was performed for each texture parameter with the Student’s t-test in the case of a normal distribution of variables, while the Wilcoxon-Mann-Whitney test was con-ducted in the presence of a non-normal distribution. Shapiro Wilk test was used to test the normality of distribution of the features. For each variable that showed a significant difference (*p* < 0.05), ROC curves were drawn, as well as a multiparametric ROC curve with all the significant features together by the de Long method. The statistical analysis was carried out using MedCalc and R (www.r-project.org) software.

## Results

The present study involved 37 patients referred to the Oncological Multidisciplinary Group of Padova (Endocrinology, Endocrine Surgery and Oncology divisions), treated with complete EDP-M regimen from 2007 to 2022. Initially, the possible presence of adequate CT imaging in the local database was evaluated, considering both timing (within one month before EDP-M treatment and within three months after the end of the chemotherapy cycle) and study protocols (requiring at least the venous phase for chest and abdomen and reconstruction for lung parenchyma). Consequently, 20 patients were excluded. Among the remaining 17 patients, tissue measurability of the disease in both involved CT scans was evaluated, leading to the exclusion of one patient.

Finally, the study population included 17 patients, 13 women and 4 men, with a mean age at diagnosis of 47 ± 15 years, described in Table 2. Primary or debulking surgery was performed before EDP-M in 12 patients, while one patient underwent surgery at the end of the chemotherapy. Four patients were not subjected to surgery due to extensive disease and/or reduced performance status (ACC was confirmed with liver or adrenal biopsy in three cases, with endocrine and radiological assessment in one). Mitotane treatment was started in all patients within two months after surgery, in all cases before EDP (patients number 1, 2, 3, 8, 9, 10 and 12 in Table 2 presented a progression with ongoing mitotane and were treated with EDP-M).

**Table 2:** Clinical and endocrine picture of patients included in the study. Histology is based upon primary/debulking surgery (indicated with complete resection = R0, microscopic residual tumor = R1 or macroscopic residual tumor = R2) or after biopsy (specified). The response to EDP was defined according to Ambrosini criteria, reassumed in materials and methods [reference number 28]. Mitotane levels indicated are those measured in two months before and after EDP

number, sex, age at EDP treatment	ACC diagnosis	Endocrine secretion	Histology, Ki67%, WEISS score and resection	ENS@T stage at diagnosis	Primary adrenal	metastatis at EDP start	start EDP (number of cycles)	EDP response	Mitotane serum level before and after EDP
1, male, 51 yrs	December 2014	not secreting	ACC, 12%, 5, R1	4	right, 24 mm	liver	January 2021 (5)	No	13.9–14 mg/L
2, female, 71 yrs	May 2019	not secreting	ACC, 30%, 8, R1	3	right, 97 mm	local	March 2020 (4)	No	15.5–15.4 mg/L
3, female, 64 yrs	September 2012	not secreting	ACC, R0	2	right, 98 mm	liver, lung	October 2014 (4)	Yes	16.2–14 mg/L
4, female, 19 yrs	September 2019	cortisol and androgens	ACC, 28%, 8, R2	4	left, 144 mm	lung, nodes	October 2019 (6)	Yes	4.4–12.7 mg/L
5, female, 67 yrs	August 2009	cortisol	ACC, R2	4	right, 80 mm	lung, nodes	September 2009 (6)	No	4.8–18.4 mg/L
6, female, 36 yrs	April 2021	cortisol and androgens	ACC, 45%, 9, R2	4	right, 11 mm	liver, lung, bone	July 2021 (6)	No	20.3–16.6 mg/L
7, male, 27 yrs	January 2016	cortisol	ACC, 10%, 6, R2	4	left, 200 mm	lung, nodes	May 2016 (6)	No	11–14.1 mg/L
8, female, 44 yrs	August 2019	not secreting	ACC, 20%, 7, R0	4	left, 140 mm	liver	June 2021 (4)	No	20.3–29 mg/L
9, male, 48 yrs	February 2018	cortisol	ACC, 15%, 6, R0	3	left, 50 mm	local	April 2019 (6)	Yes	10.8–13.5 mg/L
10, female, 27 yrs	June 2008	cortisol	ACC, 15%, 6, R2	2	left, 80 cm	liver, lung, bone	October 2009 (6)	No	19.6–17.5 mg/L
11, female, 48 yrs	October 2013	cortisol and androgens	not performed	4	left, 85 mm	liver, lung	November 2013 (4)	Yes	13.8–13 mg/L
12, male, 68 yrs	September 2006	cortisol	ACC, 60%, 8, R1	2	left, 80 mm	liver, lung	August 2007 (4)	Yes	7–10 mg/L
13, female, 41 yrs	August 2021	cortisol and androgens	ACC, 40%, 8, R1	4	left, 160 mm	liver, nodes	September 2021 (4)	No	7.2–10.5 mg/L
14, female, 57 yrs	March 2022	cortisol	ACC (liver biopsy)	4	Left, 60 mm	liver	April 2022 (6)	No	5.2–15.8 mg/L
15, female, 48 yrs	December 2021	androgens	ACC, 45%, 9 (adrenal biopsy)	4	Left, 117 mm	liver, nodes	January 2022 (4)	Yes	7.5–12.9 mg/L
16, female, 44 yrs	January 2010	cortisol and androgens	ACC, 40%, 4, R1	4	left, 150 mm	liver, nodes	June 2011 (6)	Yes	16.8–12.9 mg/L
17, female, 59 yrs	January 2020	cortisol and androgens	ACC, R2 (liver biopsy)	4	right, 175 mm	liver, nodes	February 2020 (4)	No	11.7–18.4 mg/L

**Fig. 1:**
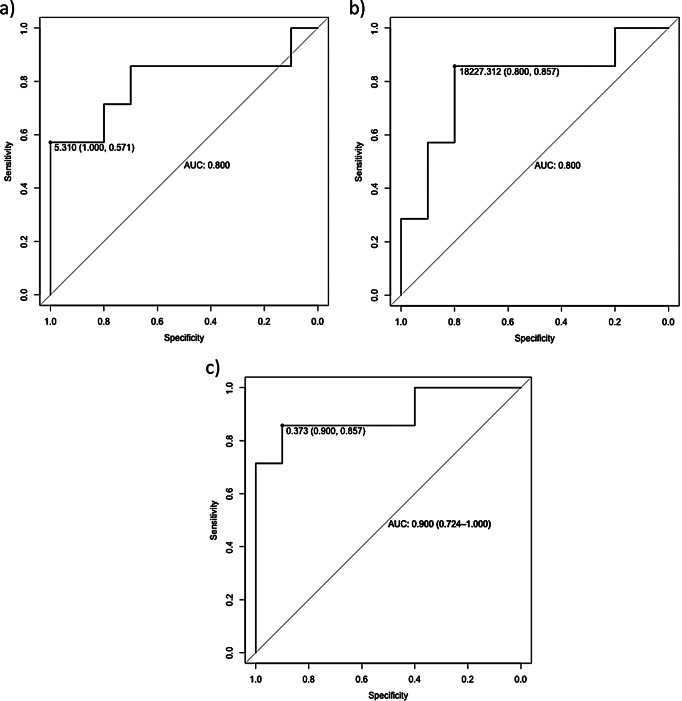
ROC curves of the selected parameters (Long Run High Grey Level Emphasis and the Intensity Histogram Kurtosis, respectively panel **a** and **b**) and combination of both features in a multiparametric ROC curve (panel **c**)

The agreement between the two radiologists was very good with a Cohen’s K > 0.9 for all the measurements of the lesions (largest diameters, volumes, and mean densitometries) at staging and restaging and for classification of the response according to RECIST 1.1, 3D-EPSSG and Choi criteria. The lesion with the best overall response was considered for each patient that, therefore, was classified as responder or non-responder to chemotherapy. Thus, 10 non-responders and 7 responders were identified. Radiomic texture features, calculated with LifeX, are depicted in Supplementary Table 1. All variables in the study showed a non-normal distribution after the Shapiro-Wilk test. Two variables, the Long Run High Grey Level Emphasis (LRHGLE_GLRLM) and the Intensity Histogram Kurtosis, were different (*p* = 0.04 for both) between groups. Other textural features were similar between the two groups. Two ROC curves, one for each significant feature, were drawn (Fig. 1, panel a and b), demonstrating a diagnostic good area under the curve (AUC). If both features were used together to draw a multiparametric ROC curve (Fig. 1, panel c) the AUC raised to 0.9 (95% CI: 0.724-1.0). The accuracy was 0.88 (95% CI: 0.63–0.98), sensitivity 0.86 (95% CI: 0.42–0.99), specificity 0.90 (95% CI: 0.55-1.00), positive predictive value 0.86 (95% CI: 0.42–0.99) and negative predictive value 0.90 (95% CI: 0.55-1.0).

## Discussion

The application of big-data to the study of texture analysis enables the discovery of novel predictive factors in oncology [20]. Radiomics leverages radiological images acquired in routine diagnostic practice, using the data to perform complex mathematical calculations. The images analyzed can be composed of pixels, the conventional unit of measurement for the surface of a digital image, or voxels, the three-dimensional counterpart of pixels [30]. Each radiological image can be seen as a set of pixels that combine precise spatial coordinates and the intensity of the grayscale level. The concept of texture in a digital image, therefore, can be understood as the distribution and ratio of intensity levels in the pixels of a given ROI or VOI: texture is a characteristic of a region of interest. To describe the relationships between the intensity values of the grayscale in the images, statistical models are generally used, employing dedicated tools and software [8]. The radiomic data thus obtained are classified into four main categories: data related to the shape of the object under examination, first- and second-order statistical data, and higher-order statistical data [8, 31].

The present study aims to determine whether texture parameters derived from CT scans of the target lesion with larger dimensions in patients with advanced ACC before EDP-M treatment are independently associated with the response to chemotherapy (response defined by two radiologists based on the simultaneous agreement of RECIST, Choi, and volumetric criteria). Two parameters could discriminate between responders and non-responders patients: they have been identified after the extraction of radiomic parameters of first-and second-order for the largest primary/metastatic mass of each patient and comparing these parameters based on the binary assignment of patients (assignment defined based on the aforementioned gold standard). This was observed both when considered individually and simultaneously.

To fully understand the clinical significance of these findings, it is necessary to delve into the meaning of the two parameters under examination. Histogram Intensity Kurtosis measures the weight of the tails of the distribution in terms of frequency, defining them as “heavy” or “light” compared to a normal distribution and how much differs from it [29]. Specifically, distributions with low kurtosis tend to have light tails and thus few outliers (interpreted as anomalous values, distant from the other available observations), conversely, higher kurtosis implies greater importance of the tails of the distribution in terms of frequency. In a normal (Gaussian) distribution, kurtosis has a value of 3. Kurtosis excess, with respect to a normal distribution, is the value k – 3. Often, kurtosis is referred to k excess (thus k − 3), hence the k excess of a normal distribution is 0. A distribution with k > 3 (i.e., k excess > 0) is called leptokurtic, while one with k < 3 (k excess < 0) is called platykurtic. The Long Run High Grey-Level Emphasis (LRHGLE) is a parameter derived from the Grey-Level Run-Length Matrix (GLRLM), which is a texture feature that provides spatial information about the distribution of gray values in ROIs or VOIs, which are defined as sets of pixels (or voxels). The GLRLM is calculated for 13 different directions in 3D (or 4 in 2D), and for each of the eleven texture indices derived from it, the 3D value is the average calculated across the 13 directions (in 3D) and the 4 dimensions (in 2D) [29]. The two elements (i, j) of the GLRLM correspond to the number of homogeneous runs of voxels (j) with intensity (i) in an image. A “run” refers to the length of consecutive voxels that share the same gray level intensity along a specific direction. The GLRLM provides higher-order statistical information and expresses how frequently in the image it is possible to observe these sequences of elements with a certain length and intensity [30]. By utilizing the GLRLM, the distribution of runs of gray levels is described, where the length of the run (run-length) measures contiguous gray levels along a specific orientation.

**Fig. 2:**
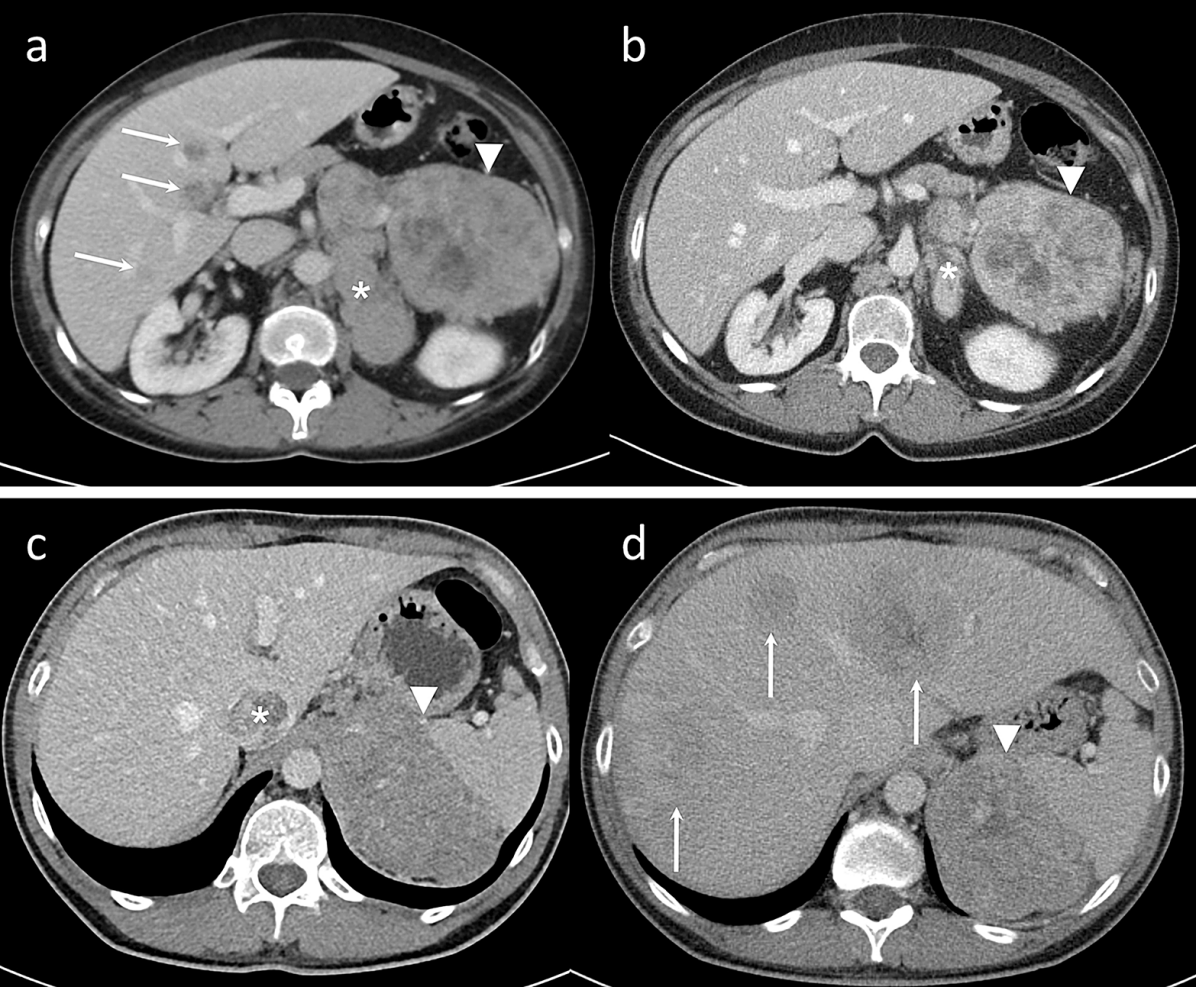
Contrast enhanced CT scans in venous phase EDP-M response in homogeneous and heterogeneous ACC. Responder patient: panel **a**) staging scan showing a large left adrenal lesion (arrowhead) with coarse and inhomogeneous texture (with high values of GLRLM_LRHGLE and Kurtosis), large loco-regional nodal metastases (asterisk) and liver metastases (arrows); panel **b**) restaging scan after EDP-M therapy with a reduction of the adrenal lesion (arrowhead) and of the locoregional metastases (asterisk) and disappearance of the liver metastases. Non-responder patient: panel **c**) staging scan showing a large left adrenal lesion (arrowhead) with fine and homogeneous texture (with low values of GLRLM_LRHGLE and Kurtosis) and a thrombus in the inferior vena cava (asterisk); panel **d**) restaging scan after EDP-M therapy with a slight reduction of the adrenal lesion (arrowhead) and the disappearance of the inferior vena cava thrombus but with the comparison of multiple liver metastases (arrows)

Therefore, by characterizing the pixels (or voxels) of the tumor, the GLRLM can provide information about the regional heterogeneity of the tumor. Specifically, finer textures tend to have shorter runs, while coarser textures will exhibit longer runs. This matrix allows for the estimation of numerous descriptors, and in our specific case, GLRLM_LRHGLE represents the value of the distribution of long runs with high grey-level intensity. The two parameters that discriminates between responders and non-responders had a higher value in the responder’s group. Therefore, patients who will respond to chemotherapy present a higher level of kurtosis of the histogram of the distribution frequency of grayscale values compared to non-responders. At the same time, they exhibit longer runs of high grayscale intensity voxels, meaning a coarser and more intense tissue texture. Although these two parameters only partially describe the characteristics of both the curve of the frequency distribution of grayscale intensities and their regional relationships in space, the underlying trend favors a coarser and more heterogeneous tissue texture in responder patients compared to non-responders (two example cases are shown in Fig. 2). At the best of our knowledge, in literature there are no studies on CT texture analysis and response to chemotherapy of ACC. In oncological imaging, several studies have highlighted the relationship between tissue heterogeneity features and the malignancy of the lesion, the probability of tumor recurrence, the reduced therapeutic response rate, and a poor prognosis [11, 32, 33]. However, contradictory results have also been found in studies on both primary colorectal cancer and metastatic cancer [32], as well as in a study by Yun et al. that analyzed radiomic data derived from contrast-enhanced CT scans in patients with pancreatic cancer. They reported an association between features suggesting more homogeneous textures and a poorer therapeutic response, suggesting a more aggressive tumor behavior [34]. Our study therefore confirms similar results: patients with advanced and metastatic ACC who would be more likely to respond to EDP-M therapy exhibited texture features consistent with greater heterogeneity and coarseness of tissue texture. Furthermore, the coarseness of the texture itself, with high-intensity grayscale voxels, may reflect a faster metabolism and cell growth, which in turn could be the basis for a better response to chemotherapy. To conclude, the correlation between these CT texture features and treatment response could suggest the future use of texture analysis as a prognostic biomarker in metastatic ACC patients.

We can also speculate that a greater tissue heterogeneity in responders may be secondary to a mitotane effect, as shown by both the higher presence of outliers in the distribution of tissue grayscale levels (due to more prominent areas of cellular necrosis) and the presence of longer runs of high-intensity gray-scale in the texture, or to a coarse presence of more functionally active cell populations that may then respond better to the EDP-M regimen.

The retrospective design, the limited number of paired patients available, and their different clinical histories (in terms of staging, mitotic index, EDP-M treatment after mitotane progression) are the main limitations of our study. Moreover, in some cases, histological confirmation was not available (as in patient number 11, with severe Cushing’s syndrome and virilization that allowed us to diagnose metastatic ACC) or the Weiss score was not calculated if surgery was performed in another center, in case of insufficient material (due to large amount of necrotic tissue), or if specimen obtained with a biopsy was not sufficient to calculate the score. A multicenter study, with a larger number of recruited patients, will be useful to consider if some known prognostic markers (tumor stage, resection, Ki-67) can be combined with radiomic to enhance its predictive role.

Even if it can be perceived as a weakness, we believe that comparing the evaluations of two radiologists provided us with a stronger standard of reference for classifying treatment response. The high Cohen’s Kappa obtained showed that the measurements by the two radiologists were reproducible. This is important because literature reports that in cases of complex tumor shapes, the measurements of the maximum axis, densitometry, and volume of the lesions can vary significantly among readers [35].

It should be underscored that the standard of reference we used required us to dichotomize the patients into responders and non-responders, without considering mixed responses to chemotherapy or the different biological behaviors of ACCs. We cannot exclude that this classification could have brought to biases in the correlation with the textural features. Nevertheless, we believe this was the most effective standard for this type of evaluation, because obtaining a histological specimen from all lesions was not feasible.

In conclusion, the higher value of kurtosis and the presence of long runs of high gray-scale intensity, obtained from the texture analysis of primary or metastatic ACC lesions in routine CT scans, may predict the response to EDP-M therapy in patients with advanced and/or metastatic ACC and could be used as a prognostic biomarker. Actually, peculiar molecular alteration profiles identified in ACC may represent new targetable events, with new or existent drugs [36]. However, if validated by further studies, radiomics and texture analysis could be used as an independent parameter to predict the response to chemotherapy in patients with advanced ACC.

## Electronic supplementary material

Below is the link to the electronic supplementary material.

Supplementary Material 1

## Data Availability

All data generated or analyzed during this study are included in this published article, dataset is available in case of reasoned request.
