# Supplementary material for: Texture analysis can predict response to etoposide-doxorubicin-cisplatin in patients with adrenocortical carcinoma
Source: J Endocrinol Invest. 2024 Oct 9;48(3):711–20. doi: 10.1007/s40618-024-02476-2 (PMC11876227; doi:10.1007/s40618-024-02476-2)
Supplement: Supplementary file 1 — Supplementary Material 1 [file 40618_2024_2476_MOESM1_ESM.pdf]

Supplementary Table 1

Radiomic Texture Features calculated with LifeX

(C Nioche, F Orlhac, S Boughdad, S Reuzé, J Goya-Outi, C Robert, C Pellot-Barakat, M Soussan, F Frouin, and I Buvat. LIFEx: a freeware for radiomic feature calculation in multimodality imaging to accelerate advances in the characterization of tumor heterogeneity. Cancer Research 2018; 78(16):4786-4789)

Intensity_based_P25	P25 corresponds more or less to the first quartile (Q1). It is defined as the middle number between the smallest number and the median of the data set
Intensity_based_P75	The 75th percentile corresponds to the third quartile (Q3). It is the middle value between the median and the highest value of the data set
Intensity_based_TLG	is the Total Lesion Glycolysis defined as the product of SUVmean by Volume in mL
Intensity − based_RIM	is the Radial Intensity Mean "Intensity_based_RIM_mean" of successive layers of voxels (envelope) from the outside of the region to the inside. Each layer is 1 voxel thick.<br>These envelopes are getting smaller and smaller (3D erosion of 1 voxel) up to the center of the ROI
Intensity_histogram_AUC_CSH	quantitative index of tracer uptake heterogeneity and/or heterogeneous response where lower values correspond with increased heterogeneity.
Grey level co-occurrence matrix (GLCM)	takes into account the arrangements of pairs of voxels to calculate textural indices. The GLCM is calculated<br>from 13 different directions in 3D with a δ-voxel distance (‖ −→ d‖) relationship between neighboured voxels. The index value is the average of the index over the 13 directions in space (X, Y, Z)
Neighborhood grey-level difference matrix (NGLDM)	Corresponds to the difference of grey-levels between one voxel and its 26 neighbors in 3 dimensions (8 in 2D). Three texture indices can be computed from this matrix
Grey-level run length matrix (GLRLM)	gives the size of homogeneous runs for each grey level. This matrix is computed for the 13 different directions in 3D (4 in 2D) and for each of the 11 texture indices derived from this matrix, the 3D value is the average over the 13 directions in 3D (4 in 2D). The element (i, j) of GLRLM corresponds to the number of homogeneous runs of j voxels with intensity i in an image and is called GLRLM(i, j) thereafter
Grey-level zone length matrix (GLZLM)	provides information on the size of homogeneous zones for each grey-level in 3 dimensions (or 2D). It is also named Grey Level Size Zone Matrix (GLSZM). From this matrix, 11 texture indices are computed.<br>Element (i, j) of GLZLM corresponds to the number of homogeneous zones of j voxels with the intensity i in an image and is called GLZLM(i, j) thereafter
compacity	The compacity feature reflects how compact the Volume of Interest is.<br>Compacity = $A^{3/2}$ /V<br>where V and A correspond to the volume and the surface of the Volume Of Interest based on the Delaunay triangulation.

Volume	is the volume in mesh of ROI
VoxelsCounting	is not IBSI compliant. It is the number of voxels in the ROI
ApproximateVolume	is the number of voxels multiplied by the volume of a voxel
MaxValueCoordinates	is pretty self explanatory. It indicates the coordinates of the maximum value
CenterOfMass (COMCoor)	is the center of mass coordinates of the studied ROI in voxels
WeightedCenterOfMass (WCOMCoor)	is the weighted intensity center of mass coordinates of the studied ROI in voxels
HOCMax	is the distance in millimeter between the activity Hotspot with maxValue
HOC05mLPeak	is the distance in millimeter between the activity Hotspot with 0.5 mL peakValue
HOC1mLPeak	is the distance in millimeter between the activity Hotspot with 1 mL peakValue
NormalizedHOCradiusROI	is the distance in millimeter between the normalized ROI maximum radius of the activity hotspot with maxValue
NormalizedHOCradiusSphere	is the distance in millimeter between the normalized sphere radius of the activity hotspot with maxValue
NormalizedCenterOfMassShiftMaxRadiusROI	is the distance in millimeter between the normalized ROI maximum radius activity with weighted center of mass
NormalizedCenterOfMassShiftRadiusSphere	is the distance in millimeter between the normalized sphere radius of the activity hotspot with weighted center of mass
MaxValuePerimeter2DDistance	is the closest 2D distance between maxValue voxel to the segmented tumor border
MaxValuePerimeter3DDistance	is the closest 3D distance between maxValue voxel to the segmented tumor border
Peak05mLPerimeter2DDistance	is the closest 2D distance between Peak0.5mL voxel to the segmented tumor border
Peak05mLPerimeter3DDistance	is the closest 3D distance between Peak0.5mL voxel to the segmented tumor border
Peak1mLPerimeter2DDistance	is the closest 2D distance between Peak1mL voxel to the segmented tumor border
Peak1mLPerimeter3DDistance	is the closest 3D distance between Peak1mL voxel to the segmented tumor border
MaxValueNormalizedWithRadiusSpherePerimeter2DDistance	is the closest 2D distance between maxValue voxel to the segmented tumor border divided by the mean spherical radius
MaxValueNormalizedWithRadiusSpherePerimeter3DDistance	is the closest 3D distance between maxValue voxel to the segmented tumor border divided by the mean spherical radius
Peak05mLNormalizedWithRadiusSpherePerimeter2DDistance	is the closest 2D distance between Peak0.5mL voxel to the segmented tumor border divided by the mean spherical radius
Peak05mLNormalizedWithRadiusSpherePerimeter3DDistance	is the closest 3D distance between Peak0.5mL voxel to the segmented tumor border divided by the mean spherical radius
Peak1mLNormalizedWithRadiusSpherePerimeter2DDistance	is the closest 2D distance between Peak1mL voxel to the segmented tumor border divided by the mean spherical radius
Peak1mLNormalizedWithRadiusSpherePerimeter3DDistance	is the closest 3D distance between Peak1mL voxel to the segmented tumor border divided by the mean spherical radius
MaxValueNormalizedWithRadiusRoiPerimeter2DDistance	s the closest 2D distance between maxValue voxel to the segmented tumor border divided by the mean roi radius

MaxValueNormalizedWithRadiusRoiPerimeter3DDistance	is the closest 3D distance between maxValue voxel to the segmented tumor border divided by the mean roi radius
Peak05mLNormalizedWithRadiusRoiPerimeter2DDistance	is the closest 2D distance between Peak0.5mL voxel to the segmented tumor border divided by the mean roi radius
Peak05mLNormalizedWithRadiusRoiPerimeter3DDistance	is the closest 3D distance between Peak0.5mL voxel to the segmented tumor border divided by the mean roi radius
Peak1mLNormalizedWithRadiusRoiPerimeter2DDistance	is the closest 2D distance between Peak1mL voxel to the segmented tumor border divided by the mean roi radius
Peak1mLNormalizedWithRadiusRoiPerimeter3DDistance	is the closest 3D distance between Peak1mL voxel to the segmented tumor border divided by the mean roi radius
SphereDiameter	is the diameter of sphere equal to equivalent volume (in mm)
StandardDeviation	is the standard deviation of all voxels intensity
IntensityPeakDiscretizedVolumeSought(0.5mL)	See its equivalent of 1mL
GlobalIntensityPeak(0.5mL)	See its counterpart of 1mL
IntensityPeakDiscretizedVolumeSought(1mL)	is the peak intensity of volume 1mL, based on discretized values
GlobalIntensityPeak(1mL)	corresponds to IBSI_0F91
LocalIntensityPeak	corresponds to IBSI_VJGA
